# Compound meta-optics: there is plenty of room at the top

**DOI:** 10.1515/nanoph-2024-0772

**Published:** 2025-04-24

**Authors:** Ahmed H. Dorrah

**Affiliations:** Department of Applied Physics and Science Education, Eindhoven University of Technology, Eindhoven, The Netherlands

**Keywords:** nanophotonics, metasurfaces, diffractive optics

## Abstract

Metasurfaces have been widely exploited in imaging and sensing, holography, light–matter interaction, and optical communications in free space and on chip, thanks to their CMOS compatibility, versatility and compact form. However, as this technology matured from novelty to performance, stringent requirements on diffraction efficiency, scalability, and complex light control have also emerged. For instance, the limited thickness of single-layer meta-optics poses fundamental constraints on dispersion engineering and lossless transmission over large-scale devices, whereas in-plane symmetry limits the polarization transformations that can be realized. Cascaded and multi-layer flat optics can alleviate these constraints, offering new possibilities for realizing high-efficiency devices, full polarization control, and achromatic response. In this perspective, recent advances in multi-layer metasurfaces including inherent challenges and opportunities will be discussed. Compound meta-optics hold the promise for enabling complex optical systems with enhanced performance and unprecedented functionality for a diverse set of applications in sensing, imaging, high-capacity communications, and beyond.

## 1 Introduction

**Figure 1:**
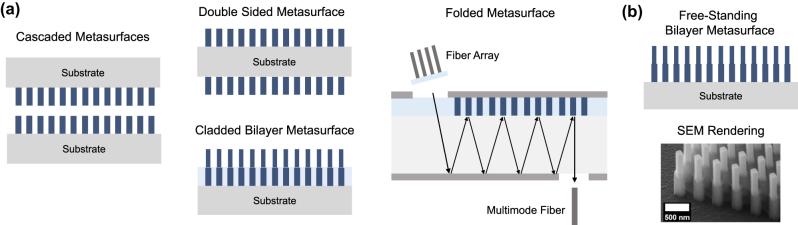
Different configurations of multi-layer metasurfaces include: (a) cascaded metasurfaces, metasurface doublets, cladded multi-layer metasurfaces, folded metasurfaces, and (b) free-standing metasurfaces with high index contrast. An SEM image of a free-standing bilayer metasurface is shown at the bottom of (b), reproduced from [107].

Optical metasurfaces represent a wide class of planar nano-optics, patterned with sub-wavelength resolution, to control light’s phase, intensity, and polarization profile, point-by-point, over a compact thickness [1], [2], [3], [4], [5], [6], [7], [8], [9], [10], [11]. With a single lithography step, a rich gamut of meta-atoms can be tailored at the nanoscale, allowing a versatile set of functionalities to be achieved using the same planar form factor [12], [13], [14], [15], [16]. To this end, flat optics has enabled a variety of applications, ranging from focusing [17], [18], [19], [20], [21], [22], [23], computational imaging [24], [25], [26], [27], [28], holography [29], [30], polarization imaging [31], [32], in addition to multifunctional [33], [34], [35], [36] and reconfigurable devices [37], [38], [39], [40], [41], [42], [43], [44], [45], [46], [47]. Thanks to their well-established CMOS-compatible nanolithography, deposition and etching techniques [48], [49], [50], [51], metasurfaces can be deployed in free space or integrated on chip [52], [53]. Although metasurfaces have historically evolved as planar photonic components [54], [55], [56], [57], [58], [59], [60] that alleviate the fabrication complexity of 3D metamaterials [61], [62], [63], [64], [65], [66], while approaching its versatility, it has become clear that their limited thickness poses many fundamental constraints in wavefront shaping, especially at high numerical apertures, as will be detailed next. Consequently, new architectures for cascaded, multi-layer, double-sided, folded, and 3D meta-optics (Figure 1) have gained much attention recently, aided by the rapid advancements in multi-level nanofabrication techniques.

## 2 Limitations of single-layer metasurfaces

The development of compound meta-optics arose partly because a single light interaction with a flat optical surface is fundamentally restricted to a finite set of achievable wavefront transformations, often posing a compromise between diffraction efficiency, bandwidth, field of view, numerical aperture (NA), and complex amplitude modulation. For instance, it is now well established that an arbitrary wave transformation via an ultrathin metasurface (much thinner than the wavelength), for e.g., achieved with plasmonic nano-antennas, even in the prototypical case of beam steering, inherently requires balanced loss and gain across the surface to achieve unitary efficiency [67], [68], [69], [70]. This stems from local impedance mismatch between the input and output fields across the surface of an infinitesimally thin meta-optic. Such a passive and reciprocal interface only supports tangential electric currents that, due to 2D symmetry of the surface, radiate equally on both sides, hindering simultaneous lossless transmission and complete phase control, and causes undesired reflection and limited polarization conversion [67], [69].

Achieving impedance matching between the input and output fields using ultra-thin gradient metasurfaces requires at least two layers to improve the transmission level [71], [72], and three (or two in reflection) to simultaneously control the underlying phase between 0 − 2*π* [67], [73], [74], [75]. This requirement can be reduced to two layers in transmission using *nonlocal* metasurfaces by relying on extended resonances or long-range interaction between the nanopillars [76], [77], [78], [79], using Huygen’s metasurfaces with electric and magnetic polarization [80] or through incorporating bianisotropy [81], [82].

Extending the thickness of single-layer meta-optics, from ultra-thin to the wavelength-scale regime, using high index contrast dielectric meta-atoms, have relaxed the tight constraints on diffraction efficiency and complete phase and polarization control [20], [48], [56], [57], [83], [84], [85], [86], [87], [88], [89], [90]. Nevertheless, lossless complex amplitude modulation using phase-only dielectric metasurfaces still mandates the use of a compound (multi-layer) arrangements [91], [92], [93]. Furthermore, on the device level, several trade-offs still exist between bandwidth, field-of-view and NA. For instance, achieving achromatic response at high NA values, wich require steep phase gradients remains an open challenge in metalens design due to limitations imposed by its thin form factor. In principle, a metalens, being a linear time-invariant system, is subject to the delay-bandwidth product, which implies that processing more frequencies can be achieved on the expense of more travel time (delay), ultimately requiring thicker or multi-layer metasurfaces [94], [95]. Similar arguments can be made for realizing meta-optics with wide field-of-view [96], [97], [98]. In short, as Prof. David Miller has aptly explained, optics require a minimum thickness to perform a wide range of tasks [99], [100].

## 3 Compound meta-optics

More generally, there are numerous applications that cannot be met with a single optic. For example, aligning two skewed optical beams collinearly with just one mirror is impossible. Similarly, spatial mode multiplexing, which involves shaping the wavefront and redirecting the momentum of incoming light, is a task that demands adiabatic light conversion across multiple planes [101]. In general, distributing the wavefront transformation across multiple layers introduces an additional degree-of-freedom, providing beneficial redundancy in metasurface design. This redundancy can enable multifunctional devices with high diffraction efficiency, broadband performance, wide field of view, and adaptable dispersion control as will be shown. It also provides an additional knob for tunability, for instance, through dynamically changing the relative position of the metalenses within a stack. This has been previously demonstrated in bilayer Moiré varifocal metalenses [102] and Airy beam generators [103] in the visible, as well as variable zoom meta-optic triplets in the NIR range [104]. Rotary varifocal doublets and triplets of this kind have also been demonstrated in the THz regime for possible application in 6G communications [105]. Additionally, vertical integration of metasurface structures on top of non-linear crystals could provide a route for the generation and control of complex quantum states, thus enabling a compact and practical platform for the development of advanced on-chip quantum photonic information processing [106]. As metasurface technologies have matured, the demand for these advanced functionalities has led to the exploration of more complex configurations, including cascaded, double-sided, and multi-layer flat optics, as depicted in Figure 1. Compound meta-optics of this nature bring many new opportunities when it comes to polarization control, dispersion engineering, and wavefront shaping as discussed next.

### 3.1 Metasurface polarization optics

Metasurface polarization optics have led to an extensive developments in vectorial holography as well as compact Stokes and Mueller imaging systems which can capture the polarization of a scene in single shot without moving parts [108]. Developments of this kind have reached a high technology readiness level (TRL) to be adopted in consumer electronics, including biometrics in handheld devices, industrial quality control, autonomous driving, AR/VR and quantum state topography [34], [109], [110].

**Figure 2:**
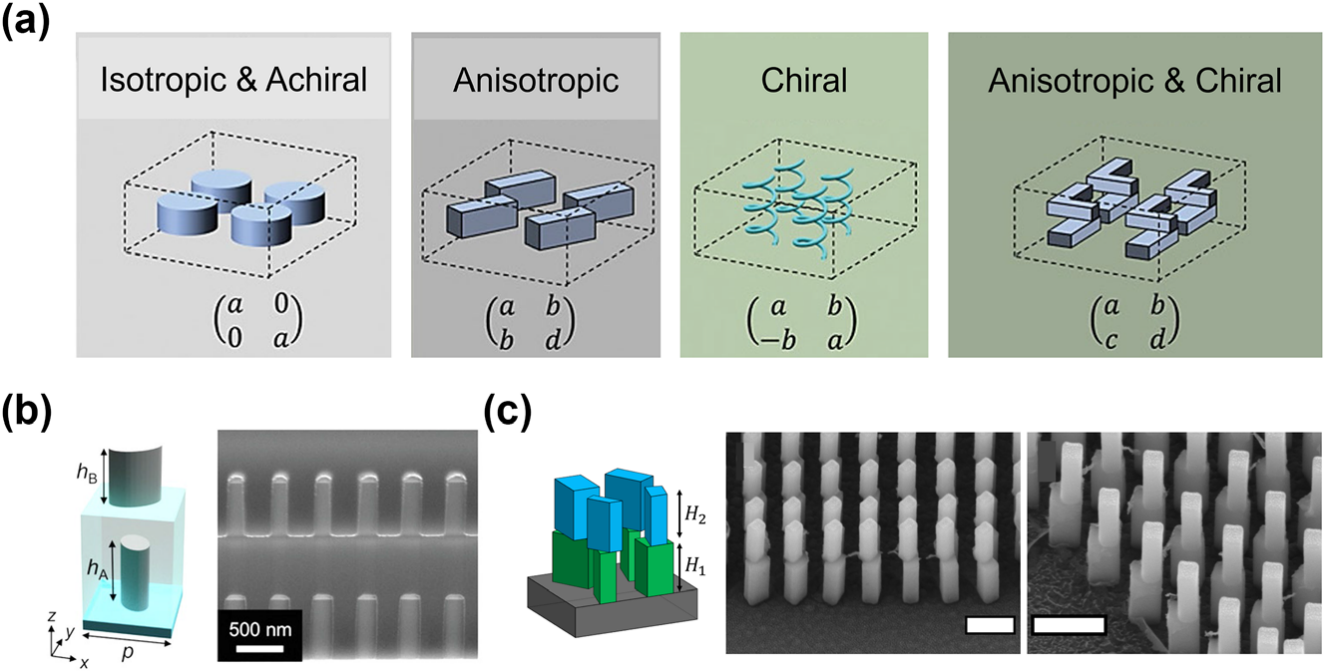
Multi-layer metasurfaces for polarization control. (a) Schematic of metasurface nanostructures and the corresponding Jones matrices that can be implemented. The letters a, b, c, and d represent complex numbers. (b) Left: Schematic of bilayer metasurface unit cell composed of 790 nm tall amorphous silicon nano posts. The bottom layer is encapsulated in SU-8. Right: SEM image of the fabricated metasurface. (c) Left: Schematic of a free-standing bilayer meta-atom made of 600 nm titanium dioxide nanofins. Right: SEM images of the fabricated device. Scale bar 500 μm. Panels (a) and (b) adapted with permission from Ref. [119]. © 2022 Wiley-VCH GmbH. Panel (c) adapted from Ref. [107].

From a more fundamental standpoint, shape-birefringent dielectric nanofins function akin to waveplates, with their transmission behavior acquiring the form of a 2-by-2 unitary and symmetric Jones matrix [87], [90], [108], [111]. While such polarization control has enabled technologies as single-shot Stokes [31] and Mueller imaging [32], unconventional polarizers [112], [113], [114], [115], and vectorial holograms [116], [117], [118], it falls short when it comes to more complex functionalities. For instance, a single-layer non-chiral nanofin cannot serve as a circular analyzer through shape-birefringence alone, as this requires the decoupling of the off-diagonal components of the Jones matrix [108]. However, rectangular non-chiral nanofins only provide linear shape-birefringence, cast as a symmetric Jones matrix. Notably, this limitation cannot be lifted with topology optimization or inverse design as it stems from the 2D symmetry of a single-layer metasurface with vertical side walls [113]. To relax this condition, bilayer metasurfaces have been introduced to enable more generalized polarization transformations by decoupling all four elements of the Jones matrix, allowing each meta-atom to impart the most general polarization transformation [119], [120], as illustrated in Figure 2. This is based on the mathematical principle that the product of two symmetric matrices (i.e., two cascaded nanofins) can form an arbitrary matrix, that is not necessarily symmetric [121].

Likewise, single-layer shape-birefringent metasurfaces can impart two different phase transformations on any pair of orthogonal polarizations, provided that the input-output polarization states are coupled [122], hindered by matrix symmetry. Cascaded metasurfaces, on the other hand, can lift this constraint by imparting arbitrary and independent amplitude and phase control on any set of two orthogonal polarizations, while completely decoupling the input and output polarization states [123], [124]. In addition to arbitrary polarization conversion, multi-layer metasurfaces also suggest new ways for wavefront shaping using Pancharatnam–Berry phase [125], [126] by allowing the input polarization to trace arbitrary trajectories in polarization space [107], [127]. This versatile control has led to a new class of geometric phase metasurfaces whose eigen polarization states are not necessarily circular but can also be linear or elliptical [107], [128].

Notably, versatile and complete polarization detection and conversion with multi-layer meta-optics can further enhance imaging systems by improving their contrast and resolution, leading to more precise sensors for industries like healthcare, automotive, and security [129], [130], [131]. In AR and VR, they enable compact devices with better 3D display quality [132], [133]. They also offer new opportunities in optical communications, energy harvesting, and quantum technologies by allowing precise and multidimensional control over light from any polarization state to another [110], [134], [135], [136], [137].

### 3.2 Dispersion-engineered meta-optics

Unlike bulk materials, whose dispersion properties are dictated by their material composition, metasurfaces offer nearly unlimited flexibility in managing dispersion by carefully designing and arranging the individual meta-atoms geometry [138], [139], [140], [141], [142], [143], [144], [145], [146]. This capability is essential in optical design, as it helps produce high-quality images by reducing chromatic aberrations, which can otherwise cause color distortion and blurriness. While methods for correcting dispersion in refractive optics have existed for a long time [147], [148], they often require combining different types of glasses, resulting in bulkier and more expensive optical systems. In contrast, dispersion-engineered metasurfaces provide an alternative route that allows for achromatic focusing using compact, planar structures, thereby minimizing size, weight, and power (SWaP) in optical systems [149], [150], [151], [152]. This has significant implications for enhancing performance in lenses, filters, and sensors. However, the performance of current metasurface platforms is ultimately limited by their thickness regardless of the sophistication of their underlying meta-atoms [94], [95].

Many adopted dispersion-engineered meta-atom configurations suffer from issues such as low transmission, narrow bandwidth, and limited polarization responses. This can, in part, be alleviated with inverse design methods such as adaptive mesh [153], [154], adjoint optimization [155], [156], [157], [158], [159], [160], [161] or deep learning [162], [163], [164] to yield more versatile free-form meta-atoms [165], [166]. Nevertheless, while it is possible to modify the phase response of meta-atoms by tuning their geometry, the imparted phase shift at the design wavelength cannot be freely controlled without affecting the phase imparted on the adjacent spectral components. In other words, for a given meta-atom height, and in the absence of resonances, it is difficult to fix the effective index which dictates the imparted phase shift (or accumulated phase delay) at a given wavelength while freely changing the effective index in the vicinity of the design wavelength (referred-to as the group delay). The degree to which phase and group delay can be controlled separately is fundamentally bound by the delay-bandwidth product [94], [95] which, at the physical layer, is dictated by the geometry, height, and composition of the meta-atom. This leads to common scenarios in dielectric metalenses where the phase coverage (or phase delay) falls short of 2*π* for a desired group delay, limiting the performance of dispersion-engineered metasurfaces over large area. Notably, inverse design techniques also become computationally intractable as the device size scales beyond a few tens of wavelengths. Moreover, adjoint methods suffer from local minima of the merit function in the design-response space, requiring multiple trials with varying starting points. Therefore, there is a critical need for new metasurface schemes that intrinsically offer high transmission, broadband, polarization-insensitive responses, without the constraints imposed by geometry.

**Figure 3:**
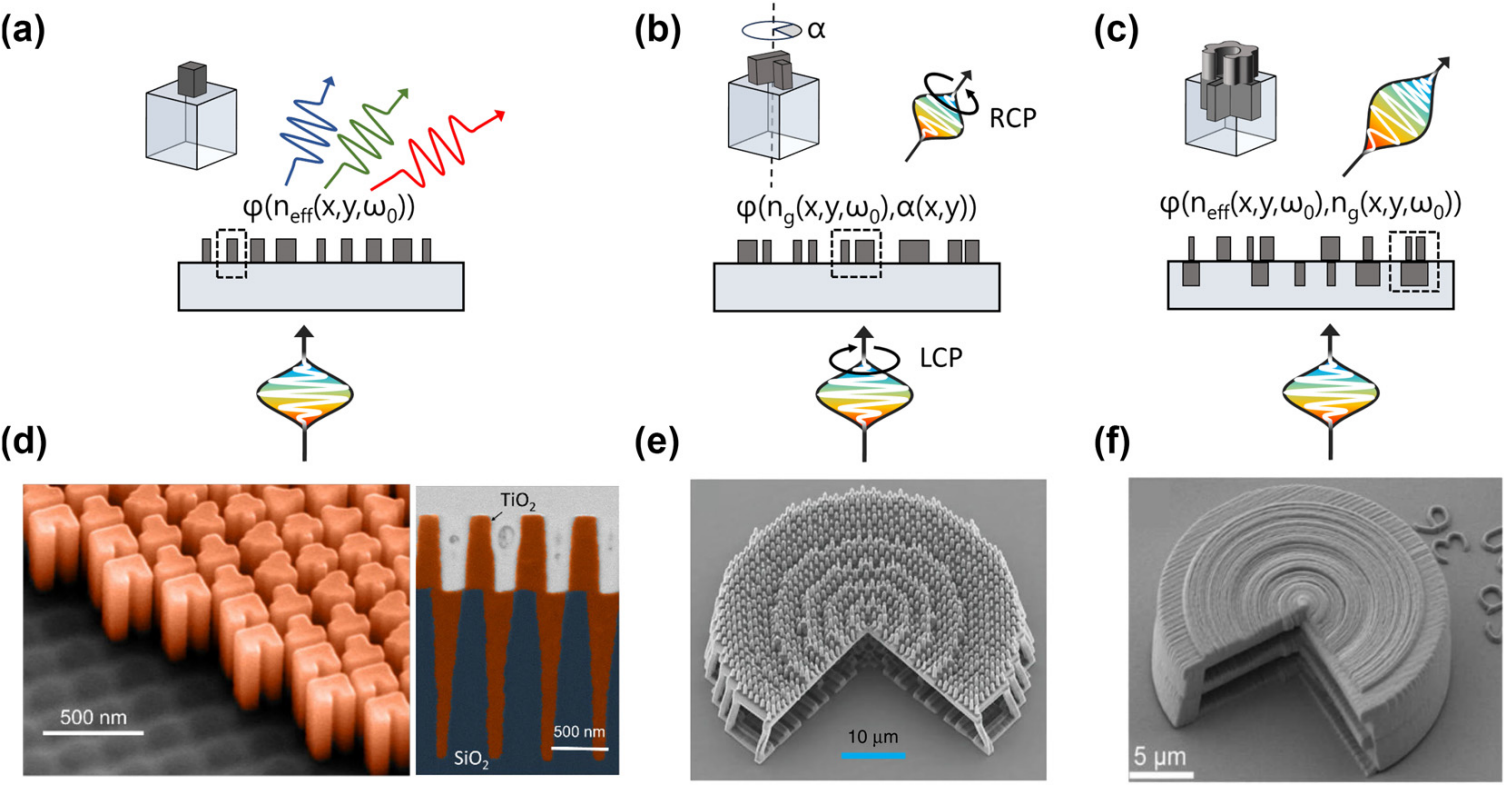
Comparison of three metasurface platforms: (a) a single-layer metasurface made up of meta-atoms with simple geometries, exemplified by a square pillar-shaped meta-atom. (b) A single-layer metasurface featuring rotated meta-atoms with varying geometries, illustrated by a double-fin meta-atom with a rotation angle *α*. (c) A bilayer metasurface created by stacking heterogeneous freeform meta-atoms. The unit cell diagram shows a freeform meta-atom stacked on top of a cross-shaped meta-atom embedded in the substrate. (d) Scanning electron microscopy (SEM) image of a fabricated heterogeneous metalens. The cross section of the stack is taken by using focused ion beam. (e) A hybrid achromatic metalens combines a phase plate and a metalens to simultaneously correct for chromatic aberration and improve focusing efficiency. SEM images of 40 μm diameter air-spaced (0.32 NA) metalens fabricated with multi-photon lithography on fused silica substrate. (f) 3D-printed multilayer achromatic metalens made from a low-index material. SEM images of the fabricated metalens operating in the visible range of 400–800 nm with 0.5 NA. Panels (a–d) adapted with permission from Ref. [172]. Images courtesy of Federico Capasso. Panel (e) reproduced from Ref. [173]. © 2020 Springer Nature. Panel (f) reproduced from Ref. [174]. © 2023 The Authors, some rights reserved; exclusive licensee AAAS. Distributed under a Creative Commons Attribution License (CC BY 4.0).

Figure 3(a) and (b) provides a conceptual visualization of the limitations encountered in single-layer metasurfaces when it comes to dispersion control. The diffractive nature of simple nanofins typically causes normally incident multichromatic light to suffer from chromatic aberrations, as a consequence of the generalized Snell’s law [1], [167], as depicted in Figure 3(a). Meta-atoms of this kind can provide full control over the phase delay, by varying the geometry of each individual nanofin, and in turn its effective refractive index. However, advanced dispersion properties, such as group delay, cannot be independently tuned using this class of devices. This sets a fundamental limitation on achieving achromatic response. To realize broadband devices, more complex meta-atoms made of coupled nanofins have been widely utilized by exploiting the resonant coupling and rotational degree-of-freedom to enable more versatile dispersion control [141], [146], [150], [151], [166], [168], [169], as shown in Figure 3(b). The group index of this meta-atom is engineered through its geometry, which determines light confinement within the nanostructure, akin to how waveguides function [85], [89]. Rotating the meta-atom by an angle *α* imparts a Pancharatnam–Berry phase of 2*α*, achieved through polarization conversion [170], [171]. However, the operation of this type of metasurfaces comes with limitations on polarization due to the intrinsic properties of the Pancharatnam–Berry phase.

As shown in Figure 3(b), a dispersion-engineered metasurface can redirect light achromatically from a left-handed to a right-handed polarization state. However, the main drawback stems from the typically low polarization conversion efficiency, which further decreases when operating away from the design wavelength [141], [146], [151], [168].

Multi-layer meta-optics holds the promise of addressing all these limitations [173], [174], [175], [176], [177]. In a first-order approximation, the phase delay and group delay of a bilayer meta-atom can be considered as the sum of the contributions from each individual layer [172]. Hence, by heterogeneously stacking two meta-atoms with different material compositions (for e.g., titanium dioxide and silica), one can significantly expand the decoupling space (independent control) between the phase and group delays, pixel-by-pixel. Furthermore, by carefully designing the meta-atoms in a freeform manner as shown in Figure 3(c), the decoupling range can be further expanded allowing for the tailored design of more advanced dispersion-engineered metasurfaces, ultimately enabling absolute focusing efficiency of ∼80% over a broadband range covering from 420 nm to NIR [172]. Metasurfaces of this type can be fabricated using a two-step nanolithography, atom layer deposition, and reactive ion etching as depicted in Figure 3(d).

Alternatively, hybrid composite metalenses comprising a phase plate and a metalens, designed to correct for chromatic aberration, have been stacked into a single element that is only a few wavelengths thick, for achromatic focusing in the NIR (1,000–1,800 nm) range [173], as shown in Figure 3(e), and more recently in the visible range (400–700 nm) by patterning the meta-atoms on top of a dispersion-matching dielectric [178]. Polarization-insensitive multi-layer achromatic metalenses have also been demonstrated in the visible by fabricating inverse designed structures in low-refractive index materials using two-photon polymerization lithography as shown in Figure 3(f).

In addition to multi-wavelength focusing with metalens doublets [176] and trilayer metasurfaces in the visible [179] and NIR [180], cascading a plano-convex and a plano-concave planar metalenses represent another clever strategy for dispersion compensation [181], elucidating the clear advantage that compound meta-optics can bring for realizing high efficiency, polarization insensitive and achromatic response. Ultra-broadband meta-optics are poised to improve imaging and metrology systems, wireless communication, and may enable innovations in fields like autonomous vehicles and wearable technologies.

### 3.3 Light routing with 3D meta-optics

Three-dimensional (3D) devices leverage a broader range of optical modes to achieve exceptionally tailored performance across various tasks, but require an efficient gradient-based optimization algorithm driven by full-wave electromagnetic simulations. Inverse design allows searching the high-dimensional space of permittivity profiles to find a local optimum for an electromagnetic merit function [156], [182], [183], [184]. By doing so, quasi-2D on-chip photonic devices have been extensively explored, where patterning occurs in the direction of light propagation within a single fabrication layer [160], [185], [186]. However, fully 3D designs for free-space applications, particularly in the infrared and visible spectra, are still in their infancy due to the increased fabrication complexity of volumetric devices. Early works in this area, which used one- and two-layer processes for optical applications or many-layer microwave prototypes, have demonstrated the potential benefits of moving to thicker devices [165], [187], [188].

**Figure 4:**
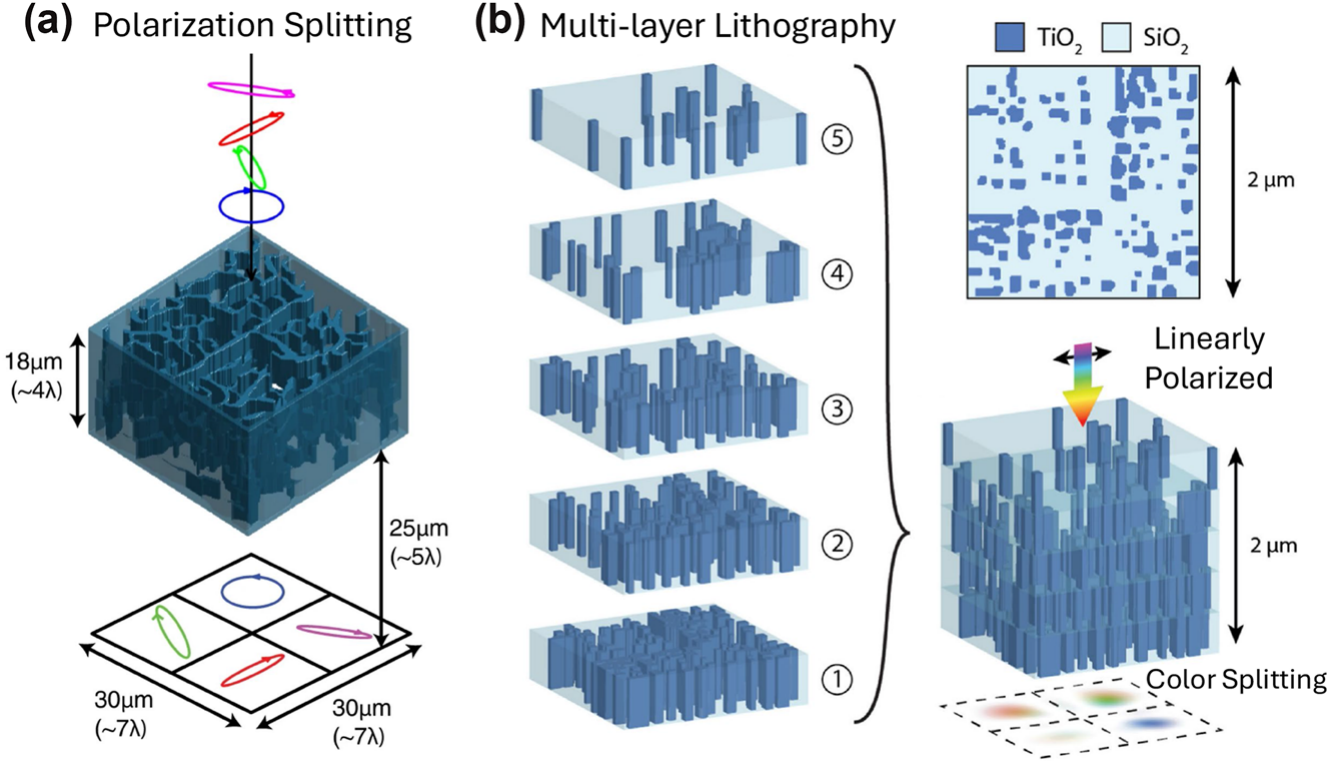
Volumetric meta-optics. (a) Schematic of full Stokes polarimetry device that sorts four polarizations; i.e., analyzes for four Jones vectors, and projects the outcome to different quadrants. (b) Spectral filter designed with multi-layer lithography. A three-dimensional scattering element created by stacking multiple two-dimensional layers. Each layer is made up of a series of titanium dioxide mesas, which can be fabricated using standard lithography and material deposition techniques. The posts are filled with silica matrix, creating a flat substrate for the subsequent layers. (c) When assembled, the five-layer stack focus linearly polarized light into distinct sensor regions based on frequency and polarization. Panel (a) adapted from Ref. [189]. © 2023 Springer Nature. Panel (b) adapted from Ref. [188]. © 2020 Optical Society of America. Used with permission.

More recently, a two-photon polymerization (TPP) lithography process has been optimized to fabricate multi-layer structures at optical wavelengths [189]. This technique has been previously used to create refractive, diffractive, gradient index, and extruded 2D inverse-designed optical components [190], [191], [192]. By exploiting the flexibility of TPP for 3D patterning at sub-wavelength resolution, several inverse-designed, multi-layer photonic devices with applications in advanced imaging in the mid-infrared band (3–6 μm) have been experimentally demonstrated [189]. This includes volumetric meta-optics for color, polarization, and vortex beam splitting using a compact footprint as depicted in Figure 4. Such development becomes particularly useful for compact imaging systems which typically deploy wavelength- and polarization-selective elements to analyze the fundamental properties of wavefronts.

In consumer cameras, for instance, absorptive filters are placed on pixel arrays to capture three or four spectral bands. The standard configuration, known as the Bayer pattern [193], arranges red, blue, and two green filters in a 2-by-2 grid. However, filtering schemes like this incur a transmission efficiency penalty because they absorb all light outside their passband, resulting in an average transmission rate of around 33 % under uniform spectral illumination. To address this issue, emerging meta-optics architectures as shown in Figure 4 can be deployed to capture light incident on a group of pixels and redirect each wavelength band or polarization to a different pixel with high diffraction efficiency [188], [194], [195], [196].

Besides light routing on chip, the development of advanced fabrication techniques for 3D printing of nano-optics will pave the path for many other inverse designed meta-optics capable of achieving high NA focusing [197], [198], asymmetric transmission [199], [200], [201], [202], [203], [204], trans-reflection [205], analogue optical computing [206], [207], [208], [209], [210], and beyond. These developments will inevitably rely on novel computational methods which enable the full-wave simulation of large scale devices and point-by-point optimization of its topology to realize the target wave transformation.

### 3.4 Multi-plane light control with folded metasurfaces

**Figure 5:**
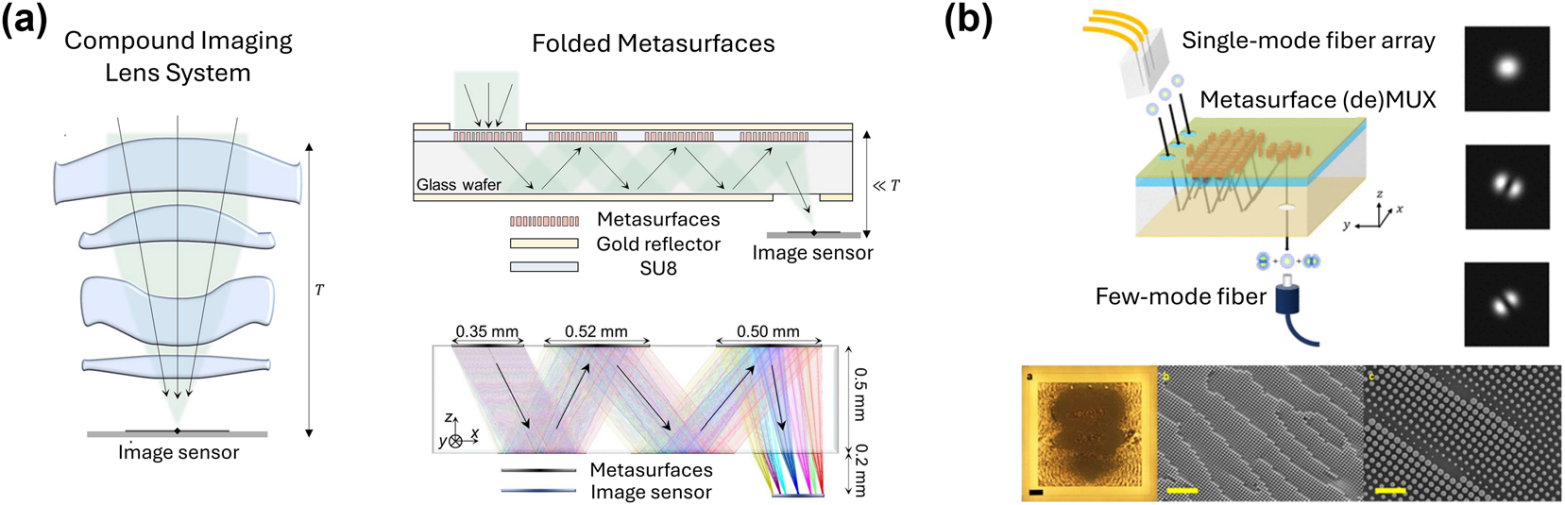
Folded metasurfaces. (a) The lens system of traditional cameras consists of multiple refractive lenses stacked vertically. The large volume of these lenses, along with the empty space between them, contributes to the overall thickness of the system. In contrast, metasurface folded optics cleverly bends the light path diagonally within the glass substrate. This approach makes efficient use of space, preserving the optical paths while significantly reducing the thickness of the lens system. The bottom right panel shows ray-tracing optimization results of the designed lens system. The colors of the rays represent different incident angles of light at the entrance aperture. (b) Schematic of a six-mode (three spatial modes and two polarization states) multiplexer based on a metasurface cavity: (a) the metasurface consists of a mirror, dielectric nanostructures with cladding, a substrate, and another mirror. Apertures on the mirrors allow light to enter and exit, with light reflecting within the cavity and coherently interfering at the output. The right column shows the measured output modes of the metasurface multiplexer. The bottom row depicts optical microscope image of the metasurface before the deposition of the gold mirror, with a scale bar of 100 μm. Scanning electron microscope images of the device without the cladding and mirror layers are also shown, with scale bar of 5 μm, and 2 μm. Panel (a) adapted from Ref. [218]. © 2024 The Authors, some rights reserved; exclusive licensee AAAS. Distributed under a Creative Commons Attribution License (CC BY 4.0). Panel (b) adapted from Ref. [101]. © 2022 The Authors. Published by American Chemical Society. Distributed under a Creative Commons Attribution License (CC BY 4.0).

The challenges associated with fabricating volumetric meta-optics can partly be alleviated by considering folded metasurfaces as depicted in Figure 5. Compound or multi-plane wavefront shaping of this kind is typically required for correcting aberrations in many optical systems but is also widely adopted for mode division (de)multiplexing where simultaneous rerouting and reshaping of the wavefront is realized using a sequence of lossless (unitary) and adiabatic transformations [211], [212], [213], [214] akin to photonic lanterns [215], [216], [217]. The compact form factor of folded metasurfaces makes them suitable for shrinking imaging systems as well as (de)multiplexers. For instance, the thickness of consumer cameras is currently dictated by the volume of its bulk refractive lens stacks and the space in between as shown in Figure 5(a). Metasurface-based folded optics can address this by guiding light along multiple folded paths within the substrate, resulting in an ultra-thin imaging system with a thickness of ∼0.7 mm, while providing quasi-diffraction-limited imaging quality at a wavelength of 852 nm [218].

A similar folded metasurface architecture has also been used in fiber-based mode division multiplexing [101]. In this work, the metasurface mode (de)multiplexer maps the fundamental mode from a fiber array to multiple spatial modes and spatially superimposes the output beam for direct coupling to a few mode fiber as depcicted in Figure 5(b). The device uses a metasurface cavity, where light propagates between a bottom mirror and an amorphous silicon metasurface-based phase mask on top.

As additional modes are added, only the lateral size of the phase mask increases, allowing scalability. This integrated design eliminates the need for free-space optics, performing collimation, focusing, beam deflection, and mode conversion on the metasurface, within the substrate’s thickness, resulting in a compact system without misalignment issues. This metasurface was optimized using adjoint analysis to minimize insertion loss, assuming light behaves as a Fabry–Perot cavity with coherent reflections. Consequently, a six-mode (de)MUX that converts three single-mode fiber (SMF) input channels (two polarization states for each) into the first three LP modes of a few-mode fiber (FMF) was realized in the C-band. This platform can potentially control additional properties like dispersion and polarization for broadband and vectorial mode manipulation as discussed in Sections 3.1 and 3.2.

The list goes on with potential applications which can make use of folded meta-optics including compact spectrometers [219], hyperspectral imagers [220], high NA off-axis illumination optics [221], augmented reality displays [222], and metasurface-stabilized microcavities [223], highlighting the range of opportunities that these meta-optics can bring in terms of miniaturization and multifunctionality.

## 4 Outlook

Although metasurfaces have been widely regarded as the planar analogue of metamaterials, the rising demand for higher diffraction efficiency, broadband operation and multifunctional response are not only difficult to realize with a single-layer metasurface from a design perspective but in many cases are not even feasible from a fundamental standpoint as highlighted throughout this article and others [67], [68], [69], [70], [94], [95], [97], [99]. Various permutations of single-layer meta-atom configurations have been exhausted over the past decade inevitably leading to a persistent trade-off between absolute efficiency, broadband operation, and large-scale devices. While it is true that inverse design and topology optimization have succeeded in pushing these frontiers by a few percentages, there is more significant advantage to be gained through vertical integration. By decomposing a complex target optical function into multiple layers, one can simplify the design requirements mandated by each layer while allowing the resulting stack to break the fundamental limits imposed on each constituent layer.

A paradigm shift in meta-optics which moves the complexity from the sophistication of precisely crafted free-form single-layer meta-atoms to “more forgiving” multi-layer stacks of more primitive building blocks will provide a more straight forward path to decoupling the otherwise entangled optical properties (for e.g., effective index, phase delay, group delay…etc.). This will also accelerate the adoption of techniques such as nanoimprint lithography for creating multi-layer stacks of large area meta-optics without the stringent requirements on deep sub-wavelength patterning. It also facilitates the hybrid integration of (conformal) meta-optics with refractive optics, thereby achieving the best of both worlds [224], [225], [226], while ensuring backward compatibility with existing optical systems by allowing meta-optics to be integrated in larger optical systems such as complex wafer metrology, ranging, or imaging systems. This will especially be suited for performing a tailored task which otherwise cannot be performed by merely relying on refractive optics; for e.g., dispersion engineering [144] and polarization control [108] are especially two key areas which can benefit from such a hybrid integration.

Furthermore, multi-layer stacking inspires alternative meta-optics schemes which leverage the capabilities of local and nonlocal metasurfaces, simultaneously, by integrating the two [227]. By combining the extended resonances enabled by neighbor-to-neighbor interactions of nonlocal metasurfaces with the local phase and polarization control of gradient metasurfaces, lossless and multifunctional wavefront shaping can ultimately be achieved. Realizing multifunctional meta-optics with less than 3–5 % reflection will mark a new era where metasurfaces can be real contenders to existing refractive optics, especially in microscopy and imaging applications which are highly sensitive to zeroth-order optical distortion.

Additionally, the integration of metasurfaces and nanoscale emitters enable access to both weak and strong coupling regimes, thereby enhancing photoluminescence, nanoscale lasing, controlled quantum emission, and exciton-polariton formation. New metasurface designs, such as surface-functionalized, chemically tunable, and multi-layer hybrid metasurfaces, offer potential applications in photocatalysis, sensing, displays, and quantum information [228]. Compound meta-optics will also allow a more direct path to tunability. By strategically co-designing a passive metasurfaces, with complex phase profile, and a less sophisticated active metasurface, a hybrid stack which achieves efficient, versatile and dynamic wavefront shaping can readily be implemented.

From a more fundamental standpoint, while many configurations of multi-layer metasurfaces can be treated as a stack of decoupled single layers, long-range interaction between the vertically stacked meta-atoms along the propagation direction can also lead to interesting phenomena related to light transport [229], [230] and disorder [231] which represent untapped potential of diffractive optics by unlocking numerous degrees-of-freedom which can ultimately be harnessed for optical computing, neuromorphic computing and beyond [208], [209], [232], [233], [234], [235], [236], [237], [238].

The prospect of realizing multi-layer flat optics to serve all these applications at scale hinges on recent advances in nanofabrication including heterogeneous integration of meta-atoms [172], [239], nanoimprint lithography [240], two-photon polymerization [189], thermal scanning probe lithography [241], [242], and roll-to-roll metasurfaces [243], [244] which are poised to enable the patterning of compound meta-optics on curved substrates for the correction of off-axis aberrations [245]. With the abundance of these tools, optical metamaterials are within reach, the future of flat optics is 3D and *there is plenty of room at the top*.
